# Mass Spectrometry Amyloid Typing Is Reproducible across Multiple Organ Sites

**DOI:** 10.1155/2019/3689091

**Published:** 2019-01-31

**Authors:** Dusan Holub, Pavla Flodrova, Tomas Pika, Patrik Flodr, Marian Hajduch, Petr Dzubak

**Affiliations:** ^1^Institute of Molecular and Translational Medicine, Faculty of Medicine and Dentistry, Palacky University Olomouc, Hnevotinska 5, 779 00 Olomouc, Czech Republic; ^2^Department of Clinical and Molecular Pathology, Faculty of Medicine and Dentistry, Palacky University Olomouc, Hnevotinska 3, 779 00 Olomouc, Czech Republic; ^3^Department of Hemato-Oncology, University Hospital Olomouc, I.P. Pavlova 185/6, 779 00 Olomouc, Czech Republic; ^4^Cancer Research Czech Republic, Hnevotinska 5, 779 00 Olomouc, Czech Republic

## Abstract

We have determined patient's amyloid subtype through immunohistochemical and proteomic analyses of formalin-fixed, paraffin-embedded (FFPE) tissue samples from two affected organs per patient. Amyloid typing, via immunohistochemistry (IHC) and laser microdissection followed by the combination of liquid chromatography with mass spectrometry (LMD-LC-MS), was performed using tissue samples of the human heart, liver, kidney, tongue, and small intestine from 11 patients, and the results were compared with clinical data. LMD-LC-MS correctly typed AL amyloidosis in all 22 FFPE tissue samples despite tissue origin. In contrast, IHC was successful only in the analysis of eight FFPE tissue samples with differences between the examined organs. In the majority of LMD-LC-MS typed samples, the level of IHC staining intensity for transthyretin and serum amyloid A was the same as that for Ig *κ* and Ig *λ* antibodies, suggesting low Ig *κ* or Ig *λ* antibodies reactivity and the additional antibody clones were essential for correct typing. Both methods used in the study were found to be suitable for amyloid typing, although LMD-LC-MS yielded more promising results than IHC.

## 1. Introduction

Amyloidosis is a rare disorder characterized by the abnormal extracellular deposition of misfolded amyloid proteins in various organs. These proteins polymerise into insoluble fibrils, with a characteristic *β*–pleated sheet structure, and other components (such as apolipoproteins, glycosaminoglycans, and serum amyloid P protein), which stabilize the fibrils to form amyloid. Amyloid accumulates in various tissues, resulting in disorganisation, damage, and organ failure [1]. Amyloid deposition can be systemic (more frequent) or localized at specific sites (less frequent), and amyloidosis can either be acquired or inherited [2, 3].

The most frequent type of amyloidosis is AL amyloidosis, characterised by the deposition of amyloid fibrils of the immunoglobulin light chain (AL *κ* or AL *λ*). AL amyloidosis is a systemic disease that is classified as a plasmacellular dyscrasia and in rare cases is associated with lymphoproliferative disorders [4, 5]. Amyloidosis derived from transthyretin (ATTR) is another common type, which results from the misfolded wild-type or mutated transthyretin (TTR) protein [1]. Chronic infections and autoimmune inflammations with increased levels of serum amyloid A (SAA) protein may result in AA amyloidosis. Moreover, mutations in the proteins, such as fibrinogen *α*, apolipoprotein A-I, apolipoprotein A-II, apolipoprotein A-IV, and lysozyme can lead to the hereditary systemic form of amyloidosis [6–8].

Up to date, there are 36 known extracellular fibril proteins that can cause amyloidosis in humans and are linked to the specific type of the amyloid disease [6]. Available treatment modalities are dependent on the particular type of amyloidosis, and therefore, an accurate diagnosis is essential. Clinically, the presence of amyloid deposits is at the first verified using histochemical staining methods, including Congo red (CR), Sirius red (SR), or metachromatic staining, during the histological examination of tissue samples obtained from an affected organ. CR staining, the standard technique for amyloid diagnosis, was developed by Puchtler et al. [9] and subsequently modified by Linke [10]. Amyloid fibrils with *β*–pleated sheet structures bind to CR dye, resulting in green, yellow, or orange birefringence under polarized light [7]. Once the amyloid has been identified, detailed characterization and typing are performed.

Amyloid typing is typically conducted via immunohistochemistry (IHC) and immunofluorescence (IF) analysis of formalin-fixed paraffin-embedded (FFPE) and/or the native frozen fixed tissue samples [10]. However, IHC often yields inconclusive results, because the antigenic epitope may be lost during FFPE tissue preparation and contamination of samples by serum proteins can result in high background staining [11, 12]. Additionally, several antibodies are required for precise determination of the most frequent amyloid protein. Differences in sensitivity and specificity of the individual antibodies may further lead to misinterpretation of the data [13].

Nowadays, laser microdissection (LMD) followed by liquid chromatography (LC) combined with mass spectrometry (LMD-LC-MS) is the typical advanced proteomic approach for the correct diagnosis and typing of amyloidosis [14–17]. LMD-LC-MS enables determination of complete protein composition and identification of the most abundant amyloid proteins from a minimal number of tissue samples [18].

In the present study, we used IHC and LMD-LC-MS for amyloid typing of 22 FFPE tissue samples. Tissues obtained from different organs were compared and the advantages and failures of both methods for the diagnosis of amyloidosis were summarized.

## 2. Materials and Methods

### 2.1. Sample Collection

For the study, we have selected twenty-two FFPE samples of eleven previously diagnosed amyloidosis cases (University Hospital in Olomouc, Department of Hemato-Oncology) in the pathology archive (Department of Clinical and Molecular Pathology, Faculty of Medicine and Dentistry, Palacky University Olomouc), where at least two different tissue samples with amyloid deposits were obtained by routine autopsy examinations. The selected patient group consisted of eight men and three women, at ages ranging from 49 to 84. The median age of these patients was 69. Samples for IHC and LMD-LC-MS were prepared from two different organs (including the myocardial, liver, kidney, tongue, and small intestine tissues) per case under the same laboratory procedures. All procedures performed in the study involving human participants were in accordance with the ethical standards of the Palacky University and University Hospital in Olomouc and/or national research committee and with the 1964 Helsinki declaration and its later amendments or comparable ethical standards. For this type of study formal consent is not required.

### 2.2. Histology and Immunohistochemistry

Three-micron thick sections of FFPE tissue were prepared for histological examination. Histological examination was performed using CR and SR staining to visualize the amyloid deposits. IHC was performed after deparaffinization, but preceded endogenous peroxidase blocking and heat-mediated antigen retrieval using two antibody panels. The first panel “Basic panel of antibodies” included four antibodies against the most common systemic (AL *κ*, AL *λ*, AA, ATTR) amyloidosis: anti-human lambda light chains (Ig *λ*, FLEX polyclonal rabbit ready-to-use, dilution 1:10), anti-human kappa light chains (Ig *κ*, FLEX polyclonal rabbit ready-to-use, dilution 1:20), anti-human amyloid A (SAA, monoclonal mouse, clone mc1, dilution 1:100), and anti-human prealbumin (TTR, polyclonal rabbit, dilution 1:4000). These antibodies were all purchased from DAKO (Glostrup, Denmark). For the second panel, “Expanded panel of antibodies” we used two antibodies to help with correctly typing the amyloid class for AL amyloidosis and included anti-human kappa light chains (Ig *κ* (KRA/KUN), polyclonal rabbit, dilution 1:2000) and anti-human lambda light chains (Ig *λ* (ULI/LAT), and polyclonal rabbit, dilution 1:500). These antibodies were purchased from amYmed (Martinsried, Germany). In accordance with the semi-quantitative evaluation of IHC staining, the intensity was classified as negative (-), weak (+), moderate (++), and strong (+++).

### 2.3. Sample Preparation, LMD-LC-MS Proteomics Analysis

Tissue samples were prepared via previously described methods [14, 16, 19]. Five-micron thick sections of FFPE tissues were placed on membrane slides (Molecular Machines & Industries GmbH, Eching, Germany) and stained with CR or SR. Positive-stained amyloid deposits were dissected using a Laser Microdissection MMI CellCut (Molecular Machines & Industries, Eching, Germany) system. Three separate regions were handled in each tissue sample and each dissected specimen contained a tissue volume of at least 0.6 nL. The excised materials were collected in three individual 0.5-mL microcentrifuge tube caps (Molecular Machines & Industries, Eching, Germany) containing 35 *μ*L of a 10 mM Tris/1 mM EDTA/0.002% Zwittergent 3-16 buffer. The collected materials were then heated at 98°C for 90 min. Subsequently, the samples were sonicated in a water bath for 60 min and digested overnight at 37°C using 0.5 *μ*g of trypsin. The resulting peptides were reduced using 3 *μ*L of 0.1 M dithiothreitol (Sigma-Aldrich, Munich, Germany) at 95°C for 5 min.

The peptide mixtures were loaded onto a C18 Acclaim PepMap Nano Trap Column (Thermo Fisher Scientific, Bremen, Germany). The peptides were separated on a 75 *μ*m × 15 cm EASY-Spray column C18 (Thermo Fisher Scientific, Bremen, Germany) using a 60-min gradient of 5–35% acetonitrile in 0.1% formic acid. Eluted peptides were analyzed using an Orbitrap Elite Hybrid Ion Trap-Orbitrap Mass Spectrometer (Thermo Fisher Scientific, Bremen, Germany) operated in data-dependent mode. Full MS scans were collected in the Orbitrap at a resolution of 60,000. The ten most intense precursor ions were sequentially isolated for collision-induced dissociation, and the resulting tandem mass spectra (MS/MS) were collected in the linear ion trap. The raw data were processed by MaxQuant software [20] and the tandem mass spectra were matched against a composite protein sequence database using the search engine Andromeda [21]. This database contains protein sequences obtained from the SwissProt database selected for the human subspecies, known human immunoglobulin variant domains, known amyloid fibril protein mutations collected from literature, and common contaminants [18]. Andromeda was configured to detect semitryptic peptides from the composite database while searching for the following variable modifications: oxidation of methionine (+15.996 Da) and n-terminal pyroglutamic acid (−17.023 Da). The cut-off of the global false discovery rate (FDR) for the peptide and protein identification was set to 0.01 [16].

In all cases, a personalized proteomic profile was created that listed the MS/MS spectral counts corresponding to the proteins identified in each of the dissections. The number of spectra associated with a protein is considered a semi-quantitative measure of its abundance. In this regard, the amyloidosis type was considered the most abundant amyloid protein (Table 3, labeled italic) detected in all dissected regions.

## 3. Results

### 3.1. Clinical Features

The clinical diagnosis and characteristics of the patients are listed in Table 1. Multiple myeloma (MM) and monoclonal gammopathy of undetermined significance (MGUS) were diagnosed in three and seven patients, respectively. Clinical diagnosis was impossible in case 10. Serum paraproteinemia of light chain type IgA *λ* (cases 7 and 8), IgG *κ* (case 1), and IgD *κ* (case 2) was identified via serum protein electrophoresis and immunofixation. Increased levels of serum-free *κ* light chain (FLC *κ*) and *λ* light chain (FLC *λ*) occurred in cases 1, 2, 3, 4, 5 and 6, 7, 8, 9, 11, respectively. All patients except those in cases 8 and 9 had an abnormal free light chain ratio (FLC *κ*/*λ* ratio). Cardiomyopathy (stage Mayo 3) was diagnosed in all patients. Proteinuria with increased creatinine and urea (cases 1, 2, 3, 4, 6, 7, 8, and 9) was detected in ten patients and renal insufficiency in eight patients (data were unavailable for case 10), respectively.

### 3.2. LMD-LC-MS/MS and IHC Analysis

The results from IHC and proteomics analysis are summarized in Table 2. LMD-LC-MS analysis revealed that the Ig kappa chain C region and Ig lambda-2 chain C region are the most abundant amyloid fibril protein in the tissues examined in five (1, 2, 3, 4, 5) and six (6, 7, 8, 9, 10, 11) cases, respectively. The AL amyloidosis type occurred in all eleven cases, whereas the AL *κ* type occurred in five cases, and the AL *λ* type occurred in six cases. These results are strongly correlated with the clinical symptoms in all patients. The detailed results from the LMD-LC-MS analysis are summarized in Table 3.

**Figure 1:**
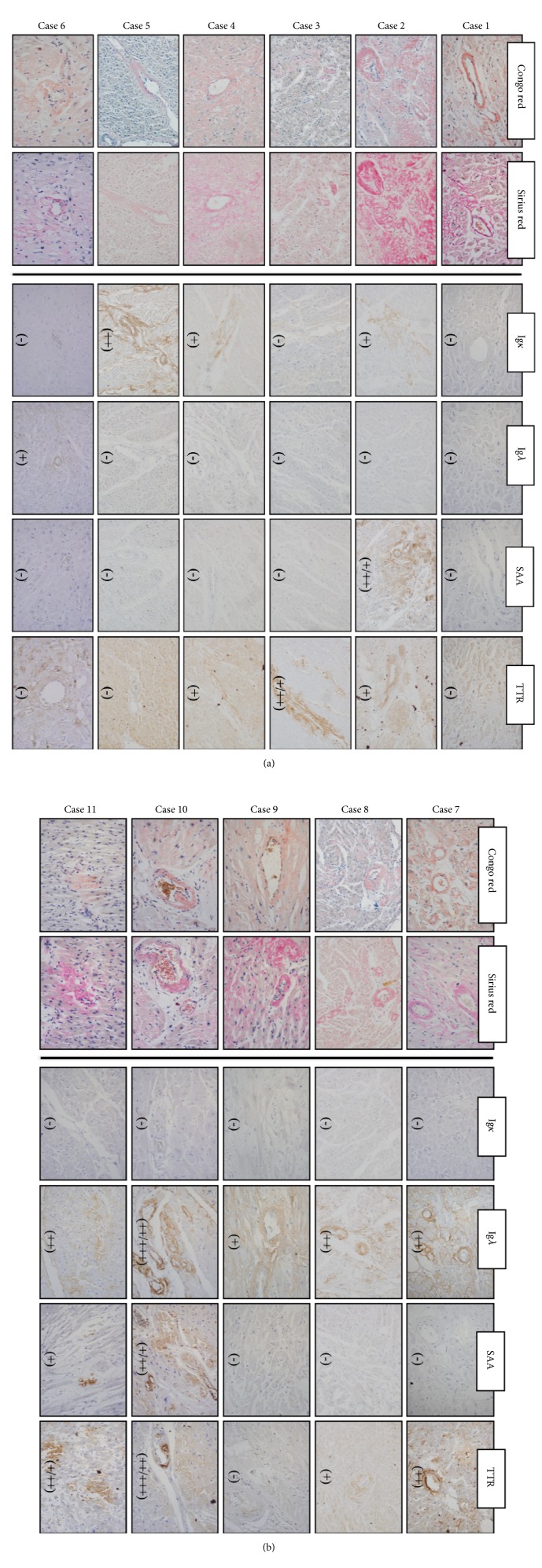
Immunohistochemical typing of amyloid in myocardial tissue by a basic panel of antibodies. The confirmation of amyloid deposition was done via Congo red and Sirius red staining, the amyloid typing via IHC analysis (right panels). The tissue sample of case 1 failed during IHC staining with AL *κ*, AL *λ*, SAA, and TTR antibodies. The examined tissues of cases 2 and 3 had a false positive reaction with SAA and/or TTR antibodies, weak and/or negative reaction with AL *κ* antibody, respectively. In cases 4, 7, 10, and 11 examined tissue had a positive reaction with more than one antibody which is classified as no immunospecific staining (NS). Amyloid fibril protein (AL *κ*) was typed correctly in case 5. Amyloid fibril protein (AL *λ*) was typed correctly in cases 6, 8, and 9. IHC staining intensity was classified as negative (-), weak (+), moderate (++), and strong (+++). The amyloid subtype was determined based on the strongest IHC reaction.

IHC analysis that used “Basic panel of antibodies” correctly typed the amyloid fibril protein in only seven specimens from four patients (both tissues in cases 6, 8, 9, and heart tissue sample in case 5). The heart tissue samples of case 1 failed during IHC staining with all four antibodies, and typing was therefore unachievable. Surprisingly, both tissues considered in case 3 had a positive reaction with the TTR antibody and negative reaction with Ig *κ* and Ig *λ* antibodies. However, clinical diagnosis and proteomic analysis typed AL *κ* amyloidosis in this case. In six other cases (2, 4, 5, 7, 10, and 11), one or both examined tissues had a positive reaction with more than one antibody. Most of the tissues had a false positive reaction with TTR (14 of 22) and SAA antibodies (6 of 22) (Figure 1 and Table 2). In three cases (nr. 1, 5, 6) a weak false positivity occurred especially in cardiomyocytes, but amyloid deposits were negative.

IHC analysis that used “Expanded panel of antibodies” (Table 2) typed amyloid fibril protein correctly in twelve specimens (both tissues in cases 4, 6, 8, 9, and heart tissue samples in cases 1, 5, 7, 11). However, analysis of the remaining ten specimens yielded inconclusive results. These examined tissues (both tissues in cases 2, 3, 10, and one tissue samples in cases 1, 5, 7, 11) had a positive reaction with more than one antibody.

## 4. Discussion

Precise typing of amyloidosis in tissues is crucial for treatment and prognosis [11]. Amyloidosis has an annual incidence of approximately 10 cases per million [22]. In 2018, the population of the Czech Republic was recorded at 10.5 million people, corresponding to an amyloidosis incidence of approximately 105 cases. In the present study, we used multidisciplinary diagnostic approaches (including clinical evaluation and biochemical tests combined with special staining (CR, SR), IHC, and proteomics analysis) for amyloid diagnosis and typing of ~10% of these cases. The clinical features of patients with amyloidosis are characterized by typical organ involvement, such as cardiomyopathy and proteinuria/RI, which are the most common organ failures in AL amyloidosis patients [2]. The data from LMD-LC-MS analysis concurred with the clinical data, where AL *κ* and AL *λ* were found to be the most abundant amyloid fibril proteins in patients with a high concentration of serum FLC *κ* and FLC *λ*, respectively (Tables 1 and 2).

Recently, IHC and LMD-LC-MS have been the main methods used for amyloid typing [10, 16]. These methods yielded different results in the current study. For example, IHC typed only eight (36%) of the 22 FFPE samples correctly, whereas LMD-LC-MS accurately identified AL amyloid fibril proteins in all 22 samples. In addition to the most abundant amyloid protein, LMD-LC-MS also identified serum amyloid P, apolipoprotein E, and apolipoprotein A-IV, which are associated with the amyloid formation in the amyloid deposits (Table 3, labeled underline) [23].

The IHC results revealed that 14 of 22 examined samples had false-positive reactions with TTR and/or SAA antibodies, where (in some cases) the intensity of IHC staining was higher than with Ig *κ* or Ig *λ* antibodies. The inadequacy of IHC for final typing of AL amyloidosis has previously been investigated [4, 24] and the critical factors affecting the performance of IHC were found to be heterogeneity of variable domains in the amino-terminal end of the light chains, the preanalytical effect of formalin-mediated tissue fixation, antigen masking due to protein folding, fragmentation of light chain molecules, and variable quality of the commercially available antibodies [4]. All these factors could decrease the reactivity of Ig *κ* or Ig *λ* antibodies. In such cases, the intensity of IHC staining could be the same for multiple amyloid proteins involved in the amyloid formation, and conclusive identification of the most abundant amyloid protein is difficult [13, 25, 26]. This difficulty was encountered in case 2 of the present study, where LMD-LC-MS identified AL *κ* as the most abundant amyloid protein. However, the intensity of IHC staining was classified as weak (+) for the Ig *κ* antibody and moderate for the TTR and SAA antibodies. In addition, the occurrence of transthyretin in the samples of case 2 was confirmed via LMD-LC-MS analysis, albeit at lower abundance than that of the Ig *κ* protein (Table 3). These results support previous findings and confirm that IHC is for amyloid typing in this case. The increased specificity of IHC can be achieved by the application of several different antibodies targeted against the same Ig *κ* or Ig *λ*, which was previously shown in several studies [4, 10]. The application of two to four antibodies against Ig *κ* or Ig *λ* led to the precise diagnosis of up to 94% of the examined cases [4, 10, 12]. Based on our IHC results from the “Basic panel of antibodies”, we decided to apply two additional antibodies “Expanded panel of antibodies” targeted against the Ig *κ* or Ig *λ*. This step helped to increase (from 31.8 to 54.5%) the specificity of the IHC staining. Nevertheless, this specificity is not definitive, owing to the small sample size, and must be further investigated.

Despite the failures, the IHC method, which can be performed without high-tech equipment, is the preferred method in most laboratories [10]. In contrast, the costs associated with LMD and LC-MS are high and therefore, these techniques are only available in specialised institutions. The LMD-LC-MS assay was, however, able to determine the correct type of amyloidosis in all cases without previous knowledge of the clinical data despite tissue origin. Taken together, both methods are essential for amyloid typing, with IHC being the first choice. However, and based on our findings, LMD-LC-MS will be useful when inconsistent clinical data are obtained from IHC, and IHC staining results in dual positive or negative outcomes.

## 5. Conclusion

In this study, we present a comparison of amyloid typing results obtained via IHC and proteomics analysis of 22 FFPE tissue samples. The amyloidosis type was correctly determined by proteomic analysis in all eleven examined cases. Considering the clinical diagnosis, we found that proteomics analysis is both very accurate and suitable for the diagnosis of amyloidosis; LMD-LC-MS method was capable of identifying a major amyloidogenic protein in all studied cases and both analysed tissue samples per case despite organ origin. In contrast, the sensitivity and specificity of IHC analysis were not so successful; just 8 from 22 samples were identified correctly in our study. This suggests that the IHC method is inadequate in many individual cases and additional analysis, including the use of multiple antibodies against the same protein, is required to improve amyloid typing.

## Figures and Tables

**Table 1:** Clinical and laboratory characteristics of patients.

Case	Sex	Age	Clinical Diagnosis	Serum Protein	FLC *κ*	FLC *λ*	FLC	Cardiomyopathy	Nephropathy
					[mg/L]	[mg/L]	*κ*/*λ* ratio		
1	M	68	MGUS	IgG *κ*	**449.5**	91.0	4.939	Mayo 3	NS + RI
2	M	58	MM	IgD *κ*	**241. 5**	1	241.5	Mayo 3	NS + RI
3	M	49	MGUS	*κ*	**2114.3**	36.7	57.61	Mayo 3	NS + RI
4	M	70	MM	*κ*	**906.3**	16.5	54.90	Mayo 3	NS + RI
5	F	78	MM	*κ*	**1588.2**	24.4	65.09	Mayo 3	NS
6	M	63	MGUS	*λ*	18.2	**593.4**	0.031	Mayo 3	NS + RI
7	F	75	MGUS	IgA *λ*	18.7	**284.7**	0.066	Mayo 3	NS + RI
8	M	84	MGUS	IgA *λ*	47.2	**84.1**	**0.561**	Mayo 3	NS + RI
9	F	67	MGUS	*λ*	21.1	**70.1**	**0.302**	Mayo 3	NS + RI
10	M	77	-	ND	ND	ND	ND	Mayo 3	-
11	M	49	MGUS	*λ*	22.6	**495.1**	0.046	Mayo 3	NS

FLC, free light chains; MGUS, monoclonal gammopathy of undetermined significance; MM, multiple myeloma; ND, not determined; NS, nephrotic syndrome; RI, renal insufficiency (creatinine level ≥130 *μ*mol/L); Mayo, Mayo Clinic staging system (1–3) based on troponin T and NT-proBNP levels.

Reference range: FLC *κ*: 3.3–19.4 mg/L; FLC *λ*: 5.7–26.3 mg/L; FLC *κ*/*λ* ratio 0.26–1.65.

**Table 2:** Amyloid typing based on IHC and proteomic analysis.

Case	Tissue	IHC: Basic panel of antibodies^a^					IHC: Expanded panel of antibodies^b^			Proteomic typing	Consensual typing
		Ig *κ*	Ig *λ*	SAA	TTR	IHC typing	Ig *κ*	Ig *λ*	IHC typing		
1	Heart	-	-	-	-	-	++	-	AL *κ*	AL *κ*	**A** **L** κ
	Liver	-	+/-	-	-	AL *λ*	+	-	NS	AL *κ*	

2	Heart	+	-	+/++	+	AA	++	+	NS	AL *κ*	**A** **L** κ
	Tongue	+	-	++	++	NS	++	+	NS	AL *κ*	

3	Heart	-	-	-	+/++	ATTR	++	-	NS	AL *κ*	**A** **L** κ
	Liver	-	-	-	+	ATTR	+	-	NS	AL *κ*	

4	Heart	+	-	-	+	NS	++	-	AL *κ*	AL *κ*	**A** **L** κ
	Liver	+	-	-	+	NS	++	-	AL *κ*	AL *κ*	

5	Heart	++	-	-	-	AL *κ*	++	+/-	AL *κ*	AL *κ*	**A** **L** κ
	Small intestine	+	-	+	-	NS	+	-	NS	AL *κ*	

6	Heart	-	+	-	-	AL *λ*	-	++	AL *λ*	AL *λ*	**A** **L** λ
	Liver	-	+	-	-	AL *λ*	+	++	AL *λ*	AL *λ*	

7	Heart	-	++	-	++	NS	++	+++	AL *λ*	AL *λ*	**A** **L** λ
	Liver	-	++	-	++	NS	++	++	NS	AL *λ*	

8	Heart	-	++	-	+	AL *λ*	+	+++	AL *λ*	AL *λ*	**A** **L** λ
	Kidney	-	++	-	+/-	AL *λ*	+	+++	AL *λ*	AL *λ*	

9	Heart	-	+	-	-	AL *λ*	-	++	AL *λ*	AL *λ*	**A** **L** λ
	Liver	-	++	-	-	AL *λ*	-	+	AL *λ*	AL *λ*	

10	Heart	-	++/+++	+/++	++/+++	NS	+	++	NS	AL *λ*	**A** **L** λ
	Kidney	-	+/++	+/++	++	NS	++	++	NS	AL *λ*	

11	Heart	-	++	+	+/++	NS	+/-	++	AL *λ*	AL *λ*	**A** **L** λ
	Liver	-	++	-	+/++	NS	-	+	NS	AL *λ*	

Ig *κ*, immunoglobulin light chain kappa; Ig *λ*, immunoglobulin light chain lambda; NS, no immunospecific staining; SAA, serum amyloid A; TTR, transthyretin; AL *κ*, amyloidosis derived from immunoglobulin light chain kappa; AL *λ*, amyloidosis derived from immunoglobulin light chain lambda; AA, amyloidosis derived from serum amyloid A; ATTR, amyloidosis derived from transthyretin.

The intensity of IHC staining was classified as negative (-), weak (+), moderate **(++**), and strong (+++). The higher IHC reaction is defining the IHC typing.

^a^Antibodies from DAKO.

^b^Antibodies from amYmed.

**Table 3:** Results of amyloid typing based on the proteomic analysis.

Case	Tissue	Biological replicates	The MS/MS counts of the most abundant amyloid proteins																
			Ig kappa chain C region	Apolipoprotein E	Ig gamma-1 chain C region	Serum amyloid P-component	Ig gamma-3 chain C region	Apolipoprotein A-IV	Ig kappa chain V-III region SIE	Ig gamma-2 chain C region	Ig kappa chain V-III region VG	Ig alpha-1 chain C region	Apolipoprotein A-I	Ig lambda-2 chain C regions	Gelsolin	Transthyretin	Ig kappa chain V-III region POM	Fibrinogen alpha chain	Proteomic typing
1	Heart	3	*51*	36	20	19	9	9	8	7	7	5	5	4	4	3	3	2	**A** **L** κ
	Liver	3	*64*	61	15	33	5	11	9	3	9	6	7	6	2	4	3	4	**A** **L** κ

2	Heart	3	*56*	25	4	10	1	37	2	1	0	1	29	2	7	11	0	11	**A** **L** κ
	Tongue	2	*44*	17	4	5	1	24	4	0	0	1	31	2	13	14	0	2	**A** **L** κ

3	Heart	3	*37*	35	2	9	1	53	1	0	0	1	15	2	2	10	0	3	**A** **L** κ
	Liver	3	*34*	62	6	9	3	26	1	0	0	1	4	2	1	9	0	10	**A** **L** κ

4	Heart	3	*107*	32	13	16	7	45	2	6	0	3	12	4	6	14	1	4	**A** **L** κ
	Liver	1	*40*	33	12	12	5	7	2	5	0	3	8	4	0	6	0	10	**A** **L** κ

5	Heart	3	*12*	12	1	5	1	12	1	0	0	1	4	1	0	2	4	0	**A** **L** κ
	Small intestine	3	*20*	11	2	13	2	38	5	0	1	4	4	2	2	2	5	2	**A** **L** κ

6	Heart	3	7	11	6	5	2	29	1	1	0	4	8	*14*	2	7	1	4	**A** **L** λ
	Liver	2	3	16	6	9	2	32	2	1	1	3	6	*12*	1	8	0	3	**A** **L** λ

7	Heart	3	6	20	7	20	3	42	2	1	0	4	17	*31*	6	8	0	10	**A** **L** λ
	Liver	2	3	15	6	27	2	31	1	0	0	3	13	*17*	1	6	0	3	**A** **L** λ

8	Heart	3	5	17	3	15	2	40	1	1	0	5	9	*25*	1	4	0	6	**A** **L** λ
	Kidney	3	11	23	9	31	4	53	2	5	1	8	8	*29*	4	10	0	4	**A** **L** λ

9	Heart	3	5	25	5	12	2	10	1	1	0	2	10	*30*	5	3	0	5	**A** **L** λ
	Liver	3	3	44	6	28	2	13	1	1	0	3	13	*30*	0	4	0	8	**A** **L** λ

10	Heart	3	10	22	6	8	3	31	0	4	0	3	15	*26*	4	3	0	4	**A** **L** λ
	Kidney	3	13	43	15	13	6	14	2	10	1	7	5	*32*	5	5	1	4	**A** **L** λ

11	Heart	3	1	14	1	7	0	31	0	1	0	1	1	*30*	2	1	0	1	**A** **L** λ
	Liver	3	1	44	0	10	0	24	0	0	0	2	0	*34*	0	2	0	9	**A** **L** λ

Italic indicates the most abundant amyloid protein in the sample and underline indicates proteins associated with the amyloid formation.

## Data Availability

The data used to support the findings of this study are available from the corresponding author upon request.
